# Influenza immunization policies: Which could be the main reasons for differences among countries?

**DOI:** 10.1080/21645515.2017.1405188

**Published:** 2017-12-21

**Authors:** Nicola Principi, Barbara Camilloni, Susanna Esposito, for the ESCMID Vaccine Study Group (EVASG)

**Affiliations:** a Emeritus Professor of Pediatrics, Università degli Studi di Milano, Milano, Italy; b Department of Experimental Medicine, Università degli Studi di Perugia, Perugia, Italy; c Pediatric Clinic, Department of Surgical and Biomedical Sciences, Università degli Studi di Perugia, Perugia, Italy

**Keywords:** influenza, influenza prevention, influenza vaccine, influenza virus, vaccination strategies

## Abstract

Despite the availability of effective prophylactic and therapeutic measures, influenza remains one of the most important infectious disease threats to the human population. Every year, seasonal influenza epidemics infect up to 30% of the population; a relevant portion of the ill are hospitalized, and more than a marginal number die. In an attempt to reduce the medical, social and economic burden of influenza, vaccines are recommended by many health authorities worldwide. However, not all countries have a national program for influenza immunization. The main aim of this paper is to list the differences among influenza immunization policies of various countries, highlighting the most important scientific reasons that may have led health authorities to make different decisions. The manuscript highlights that national influenza immunization policies can vary significantly from country to country. These differences arise from insufficient evidence of the relevance of influenza infection from a clinical, social and economic point of view. The lack of precise data on the true frequency and clinical relevance of influenza infection makes it nearly impossible to establish the economic burden of influenza. Moreover, it remains very difficult to evaluate the efficacy of the different influenza vaccines and whether their use is cost-effective considering the various types of people receiving them and the indirect advantages. Disparities among countries will be overcome only when more reliable data regarding all these aspects of influenza infection, particularly those related to the true impact of the disease, are precisely defined.

## Introduction

Despite the availability of effective prophylactic and therapeutic measures, influenza remains one of the most important infectious disease threats to the human population.^1^ Every year, seasonal influenza epidemics infect up to 30% of the population; a relevant portion of the ill are hospitalized, and more than a marginal number die. The World Health Organization (WHO) has estimated that the annual epidemics of influenza cause approximately 3 to 5 million cases of severe illness requiring hospitalization and approximately 250,000 to 500,000 deaths worldwide.^2^ The impact of influenza is even greater when occasional drifted viruses emerge, as demonstrated by the dramatic 1918 Spanish pandemic, which caused an estimated 50 to 100 million deaths.^3^

In an attempt to reduce the medical, social and economic burdens of influenza, vaccines are recommended by many health authorities worldwide, including the WHO through the Global Action Plan.^4^ However, not all countries have a national program for influenza immunization. In 2014, only 59% of the 194 WHO Member States had a national influenza immunization policy. In addition, the immunization rates were significantly higher in high-income (92%) than in low and lower-middle income countries (8–47%). Moreover, even when a national policy had been developed, details of such programs varied significantly from country to country and from WHO recommendations. Differences were found in the types of people for whom immunization was recommended, the types of vaccine that were administered and the mechanisms for funding.^5^ These differences reflect unsolved doubts regarding the clinical relevance of influenza and the need for its prevention with the presently available vaccines, together with many organizational and economic problems. Moreover, they partially explain why influenza vaccination coverage remains significantly lower than desired, even in those groups of individuals for whom epidemiological and clinical evidence strongly recommends influenza vaccination.^6-8^ The main aim of this paper is to discuss the differences among influenza immunization policies of various countries, highlighting the most important scientific reasons that may have led health authorities to make different decisions.

### Types of people requiring immunization against influenza

In some countries, such as the USA, influenza vaccination is recommended for all individuals, regardless of age and health conditions.^9^ However, in most countries that have an influenza immunization policy, vaccination is recommended only to the elderly and to subjects of any age who are considered to be at an increased risk of influenza related complications. However, although the target population appears the same for all the countries with advanced health system, the subjects for whom vaccination is recommended can be different. For example, in Europe, the cut-off age for vaccination of the elderly is 64 years in most countries, but it is 59 years in Germany, Hungary, Iceland, the Netherlands, and Slovakia, and only 54 years in Malta and Poland (Table 1).^10^ These differences are not based on real variations in biological stages among people; they are simply the consequence of a social construct that can vary culturally and historically. However, studies show that immunosenescence begins by 65–70 years of age.^11^ Therefore, if the rationale to recommend influenza vaccination is to protect immunocompromised elderly people from severe influenza, anticipating the administration of the vaccine before the age of 65 seems an excessive precaution. Furthermore, advances in medical and health science have led to a rapid increase in the average lifespan, with the persistence of normal body functions much longer than previously thought.^12^

**Table 1.** EU/EEA Member States recommendations for influenza vaccinations in the general population.

HEALTHY CHILDREN AND ADOLESCENTS
UNIVERSAL VACCINATION: Austria, Estonia, Poland
≥6–24 MONTHS: Latvia, Slovenia
≥6–36 MONTHS: Finland
≥6–59 MONTHS: Malta
≥2–4 YEARS: UK England, UK Wales
≥2–11 YEARS: UK Northern Ireland, UK Scotland
11 YEARS: UK Wales
≥6 MONTHS-12 YEARS: Slovakia
NO RECOMMENDATION: Bulgaria, Croatia, Cyprus, Czech
Republic, Denmark, France, Germany
Greece, Hungary, Iceland, Ireland, Italy,
Liechtenstein, Lithuania, Netherlands,
Norway, Portugal, Romania, Spain,
Sweden
ADULTS
≥18 YEARS: Austria, Estonia, Poland
≥50 YEARS: Belgium, Ireland
≥55 YEARS: Malta
≥59 YEARS Slovakia
≥60 YEARS: Germany, Greece, Iceland, Netherlands,Portugal
≥65 YEARS: Bulgaria, Croatia, Cyprus, Czech
Republic, Denmark, Finland, France,
Hungary, Italy, Latvia, Liechtenstein,
Lithuania, Norway, Romania, Slovenia,
Spain, Sweden, UK.

However, the most striking differences regard the list of patients to whom the influenza vaccine is recommended because of suffering from a severe chronic condition. During the 2014–2015 influenza season, all 30 Member States of the European Union that participated in a survey sponsored by the European Center for Disease Prevention and Control (ECDC) recommended seasonal influenza vaccination for patients with immunosuppression, metabolic disorders, and chronic pulmonary, cardiovascular and renal diseases. However, only 28, 27, and 19 countries recommended vaccination for people with HIV/AIDS, hepatic disease, and morbid obesity, respectively.^13^ Several reasons may explain these different policies. First of all, this happens because the association between a given underlying condition and the development of influenza-related complications may have been considered but not fully confirmed. The available data regarding the immunogenicity of and protection offered by influenza vaccines may have been considered inadequate to support vaccination. In patients with immune-mediated diseases, vaccine administration may have been associated with the risk of deterioration of the underlying clinical problem. Finally, it is possible that in some cases, the lack of attention of healthcare providers regarding influenza and its complications may have caused a delay in recruiting useful information capable of better defining vaccine use policy. Obesity is one of the best examples in this regard. Morbid obesity is a predisposing factor for the development of serious co-morbidities, such as type 2 diabetes and cardiovascular problems. Moreover, it is associated with an increased risk of infection, partly due to a slight but significant immunodeficiency.^14^^,^^15^ Both co-morbidities and immunodeficiency are *per se* risk factors for severe influenza, which explains why most national health authorities worldwide have recommended influenza vaccination for patients with these conditions for many years. However, very few countries, including the USA, have previously recommended influenza vaccination for obese subjects without clinically symptomatic co-morbidities.^16^ The policies of many countries changed only after the 2009 influenza pandemic, when it was demonstrated that obese adults could have a more severe case of influenza with increased rates of admission to the intensive care unit (ICU) than normal weight subjects, independent of the presence of other, already diagnosed, obesity-related underlying diseases.^17^ After that pandemic, the number of countries that recommend influenza vaccination to obese subjects has progressively increased, but several of those countries have still not changed their policy. Between 2102 and 2014 in Europe, the proportion of Member States recommending the seasonal influenza vaccine for obese individuals has only increased from approximately 50 to 60%.^13^

Along with the poor consideration of the data that emerged during the pandemic, another reason for the lack of recommendations is a certain degree of perplexity about the actual immunogenicity and efficacy of the influenza vaccine in obese patients. Data regarding the protection offered by this preventive measure are contrasting, although the most recent clinical trials seem to indicate that obese humans have a normal or even higher antibody production.^18^

### Recommendations for specific groups of people

Pregnant women, healthy children and health care workers (HCWs) are three groups of subjects for whom the importance of a systematic influenza vaccination has been largely discussed in recent years and for whom national policies vary significantly.

### Pregnant women

The need for the protection of pregnant women is strongly supported by those who believe that some of the physiological changes that occur during pregnancy, particularly those regarding respiratory function, leave the woman and her growing baby at a greater risk of serious influenza complications.^19^ However, this is not a universally accepted position: in 2014, among the WHO Member States, 25% of high-income countries, 50% of upper middle-income countries and 74%-96% of lower and lower-middle income countries did not consider influenza a disease for which prevention during pregnancy was necessary.^5^ In Europe, in the same influenza season, 27 out of 30 European Member States that had participated in the previously cited ECDC survey recommended influenza vaccination for pregnant women.^13^ Conversely, Bulgaria, Malta and Slovakia did not include pregnant women in the national immunization program. Moreover, among those countries recommending the vaccine, Croatia and the Netherlands considered vaccination only for pregnant women with chronic medical conditions. Differences were also evidenced in the period of pregnancy suggested for vaccination. Most of the countries recommended vaccination at any stage of pregnancy, but Belgium, Cyprus, Denmark, Germany, Italy, Norway, and Sweden indicated that the best time was during the second and third trimesters. Finally, in Germany and Norway, healthy pregnant women were considered differently from those with chronic medical conditions. In the first case, the second and third trimesters of pregnancy were suggested, whereas in the second case, the first trimester was considered the best time for immunization.^13^

In an attempt to explain these differences, most of the potential reasons already cited can be considered. First, it is possible that national immunization policies have been influenced by the opinion that a positive impact of the influenza vaccine on pregnancy has not been demonstrated. The results of *ad hoc* studies are conflicting, and in many cases, the methods used to collect data are largely questionable. It has long been thought that pregnant women were at an increased risk of severe influenza and death. This was suggested mainly by epidemiological studies including those performed during the 1918, 1957, and 1968 pandemics.^20-22^ However, several observational studies have led to different conclusions. A recently published systematic review and meta-analysis that included 152 observational studies reported that only hospitalization was more common in pregnant patients than in non-pregnant patients (odds ratio [OR] 2.44, 95% confidence interval [CI] 1.22–4.87), whereas pneumonia incidence (OR 1.80, 95% CI 0.72–4.49), ICU admission (OR 0.85, 95% CI 0.62–1.17), mechanical ventilation support (OR 1.21, 95% CI 0.70–2.08), and all-cause mortality (OR 1.04, 95% CI 0.81–1.33) were equally distributed between groups.^23^ This could be considered evidence that health care providers are prone to hospitalize pregnant women for precautionary reasons fearing complications rather than complications that really exist. Conversely, several studies included in the meta-analysis had relevant methodological problems and, as reported by the authors themselves, it remains uncertain whether these findings represent a true absence of association or whether they are a result of bias. Similar conclusions have been drawn by Katz et al. in a systematic review of the available literature.^24^ These authors reported that almost all of the studies that have evaluated the impact of influenza on pregnancy were of low quality because they lacked laboratory confirmation of diagnosis, lacked population denominators, or used ecological study methods.

A second problem that may have led decision-makers to exclude pregnant women from vaccination is the fear of severe adverse events for the mother or the fetus. These concerns of health authorities are clearly demonstrated by evidence that the information regarding the use of influenza vaccines during pregnancy, including those that are detailed in commercial products, limit or even contraindicate the administration of the vaccine in pregnant women, although they have been approved by regulatory authorities.^25^ However, in the light of the available data these concerns do not seem adequately motivated. A review of the Vaccine Adverse Event Reporting System of the USA has highlighted that this problem does not exist because spontaneous abortion, stillbirth and preterm delivery were reported with similar prevalences in vaccinated and unvaccinated mothers.^26^ Moreover, the incidence of major birth defects did not differ substantially in the babies, even when the vaccine was administered during the embryonic life.^27^^,^^28^

The exclusion of pregnant women from influenza vaccination could also depend on negative opinions about the existence of true advantages for the child. Several randomized, placebo-controlled trials have recently assessed the incidence of laboratory-confirmed influenza in children born to mothers who had received the vaccine during pregnancy. In all cases, a reduction of influenza infection in the child during the first months of life was reported. Steinoff et al., who followed children for 180 days after birth, reported a 30% (95% CI 5–48) reduction in infant influenza infections.^29^ Even better results were obtained in prospective, controlled, blinded, randomized studies conducted in Bangladesh,^30^ South Africa^31^ and Mali^32^ where reductions of 63% (95% CI 5–85), 48.8% (95% CI 11.6–70·4), and 33.1% (95% CI 3.7–53.9), respectively, were observed. Antibodies cross the placenta and reach the fetus,^33^ and the monovalent 2009 pandemic vaccine has demonstrated that antibody levels equal to or greater than the correlate of protection calculated in adults (HAI titer ≥1:40) can be achieved in 87% of infants.^34^ This is a critical point because maternal immunization is the only method that can ensure a certain degree of protection against influenza in babies younger than 6 months of age when they cannot receive any of the presently available vaccines.^35^

However, these results may have been considered inadequate to justify the recommendation in pregnant women. In some of these studies, although it was effective, the administration of a vaccine was associated with the protection of a relatively low number of children, justifying doubts on the real cost-effectiveness of the vaccination. Moreover, recent studies in which the impact of the vaccine was evaluated using laboratory confirmed influenza diagnoses have been preceded by a number of studies in which hospitalization rates or severity of the respiratory infection have been tested as indirect alternative markers of the effectiveness of the vaccine. Although debatable for the methods used, these studies frequently reported that maternal immunization was poorly or ineffective in reducing respiratory infections in the infant.^36^

In summary, conflicting information is available, and it is possible that negative results have made decision makers cautious and have led them to exclude pregnant women from recommendations. Additional incentives to wait for the results of further studies before recommending the influenza vaccine to pregnant women may have been derived from the lack of definitive data about the best moment for vaccine administration and the duration of protection in the infant. Peak antibody concentrations in the blood of the mother occur approximately 4 weeks after vaccine administration, similar to non-pregnant subjects.^37^ Maternal immunoglobulin G (IgG) concentrations in fetal blood increase from early in the second trimester through term, with most antibodies being acquired during the third trimester.^38^ This indicates that for the protection of the child, the best moment for maternal immunization is the end of the second or the beginning of the third trimester of pregnancy. However, if the target is the protection of the mother, the influenza vaccine should be given during the first trimester. Moreover, there are data indicating that protection declines with time and that at 16 and 24 weeks of age, less than 40% and less than 10.0% of children, respectively, have antibody concentrations against influenza strains higher than the minimum considered protective.^39^ Furthermore, true protection might be even lower if the hypothesis is confirmed that the correlate of protection in children is significantly higher (HAI titer ≥ 1:110) than in adults.^40^

### Healthy children

National influenza immunization policies differ significantly among various countries. The USA recommends influenza vaccination for all healthy children, independent of age,^9^ Canada considers only healthy children aged 6 to 59 months to be at risk,^41^ and recommendations in Europe vary from country to country^13^ (Table 1). Austria, Estonia, and Poland follow the same national immunization policy of the USA whereas all other countries have limitations or do not recommend influenza vaccination for healthy children. Latvia and Slovenia in particular limit vaccination to children aged ≥6 to 24 months, whereas Finland, Malta and Slovakia extend recommendations to those aged ≥6 to 36 months, ≥6 to 24 months and ≥6 months to 12 years, respectively. In the UK, England^42^ and Wales^43^ have implemented a program starting in younger children and progressively including the entire pediatric population up to 13 years old. Conversely, Scotland^44^ and Northern Ireland^10^ have decided to directly vaccinate all subjects aged 2 to 11 years. All other countries, including France, Germany and Italy, do not consider vaccinating healthy children.

Supporters of general immunization base their recommendations on two assumptions. Younger children, particularly those aged <2 years, are not thought to differ substantially from the elderly. Similar to elderly people, younger children are thought to be more susceptible to influenza infection and to suffer from more severe disease compared to older children and adults.^45^ The recommendations for school-aged children arise from the evidence that they are the most important cause of the diffusion of the infection because they spread the virus for a longer time and in greater amounts than adults.^46^

The clinical relevance of influenza in the first years of life in the pediatric population was first suggested by two well-conducted studies published at the beginning of this century. These studies showed that during the influenza season, outpatient visits, hospitalization rates and antibiotic consumption for respiratory infections significantly increased in younger healthy children.^47^^,^^48^ Despite their interest, these findings had a poor impact on influenza vaccination recommendations, and in most countries, younger children remained excluded from national immunization policies, primarily because no identification of disease etiology was determined in these studies. Consequently, it is possible that other viruses, instead of influenza viruses, could be the cause of the increased incidence of respiratory infections. However, more convincing data were collected during the 2009 influenza pandemic. Several studies in which the diagnosis of influenza was based on specific reliable laboratory tests have confirmed that infants and toddlers, even if healthy, have the highest risk of hospitalization and the highest proportion of severe respiratory cases among all monitored subjects.^49-59^ Moreover, contrary to what was previously thought, healthy children with influenza are at a high risk of death. During the four influenza seasons from 2013–2014 to 2016–2017, 452 influenza-associated pediatric deaths were reported to the CDC.^60^ As previously reported for the 2009–2010 pandemic season,^61^ many of these cases (up to 50% in some seasons) occurred in otherwise healthy children.^62^ Finally, the data from Europe showing that pediatric influenza had marginal clinical relevance and did not cause death largely underestimated the importance of the disease. This occurred because in many European countries, even in hospitalized children, influenza is diagnosed in few cases. Frequently, the etiology of respiratory infections is not confirmed by reliable laboratory tests, and even when they are performed, they frequently do not lead to an influenza diagnosis because virus shedding is no longer present. Moreover, influenza does not appear as the cause of death because complications are preferentially reported.^63-67^ When all these findings were considered, a number of European countries have modified their immunization policies and have followed the USA by including younger children in the recommendations. However, in most cases, school-aged children are presently not included because the disease in these subjects is usually mild, and poor importance is ascribed to the role the children can have in the diffusion of the infection. This can be debated because administration of the vaccine to a large portion of school-aged children is not only useful to reduce absenteeism during the influenza season,^68-70^ but it has also been associated with a significant reduction of influenza-like illness (ILI) incidence in the general population.^71-74^ Moreover, a direct relationship between the activation of influenza vaccination programs in older children and a reduction of all cause-deaths and of deaths related to pneumonia and influenza in the community was reported.^72^^,^^75^ However, most decision makers attach more importance to the fact that reaching high vaccination coverage, even through the school, can be very difficult and can cause problems with parents.^76-78^

A second problem that might explain why vaccination is not recommended in younger children despite their increased risk of severe influenza is the conviction that the available influenza vaccines are poorly effective in the first years of life. Most of the opponents to vaccination highlight the results of a meta-analysis, concluding that influenza vaccines are hardly effective in children >2 years of age and do not differ from placebo in younger children.^79^ However, these conclusions are not supported by the evaluation of some of the studied included in the meta-analysis and by the consideration of the most recent clinical trials. The meta-analysis includes some studies with a relevant risk of bias that might have led to incorrect results. Moreover, the meta-analysis was conducted before the completion of a number of well-conducted studies in which the effectiveness of vaccines was measured by diagnosing influenza only with reliable molecular biology tests.^80-83^ In these studies, a vaccine efficacy not substantially different from that usually reported for old people receiving the same vaccine was reported. If the vaccination of elderly people is considered mandatory, it is difficult to understand why the vaccine cannot be recommended for younger children, who have a similar risk of severe disease.

### Health care workers (HCWs)

Health care workers (HCWs) are the people who are directly involved in patient care and those who are potentially exposed to infections that can be transmitted to and from HCWs and patients. The CDC recommends that these individuals, irrespective of their work, receive an annual influenza vaccine.^84^ The same recommendation has been made in 2014 in Europe in 24 of the 30 Members that participated in the ECDC survey.^13^ However, in Portugal, Northern Ireland, Norway, Scotland, Slovakia and Sweden, the recommendation was only made for specific groups of HCWs, including those caring for outpatients, inpatients, and patients in long-term care. Finally, in Denmark, no national recommendation has been officially prepared, but most regions and municipalities offer HCWs free vaccinations. In all European countries, however, HCW vaccination is voluntary, and there is no penalty for not receiving the vaccine. The same is true in the USA, although attempts to oblige HCWs to receive the influenza vaccine have been made. In 2009, the state of New York established that all HCWs, with the exception of those with medical contraindications, should be vaccinated annually as a condition of employment.^85^ Moreover, despite the legal challenges triggered by this decision, similar initiatives have been taken by several medical centers, including the Virginia Mason Medical Center. The resulting increase in vaccination coverage was significant.^86^ However, the influenza vaccine is no longer mandatory in the state of New York for HCWs, although any effort to increase vaccination coverage among these individuals must be made, and it is established that any healthcare facility, residential facility and hospice must require that HCWs who are not vaccinated against influenza wear a surgical or procedure mask during influenza season while working in areas where patients may be present.^87^ The idea that health treatment cannot be mandatory has prevailed worldwide. Presently, differences among countries regarding the recommendations for influenza vaccination in HCWs are likely related to organizational and economic evaluations.

### Vaccines and immuinization policies

Differences among influenza immunization policies can derive from the characteristics of the available vaccines. Presently, two types of influenza vaccines are on the market: the parenteral inactivated influenza vaccine (IIV) and the intranasal live attenuated influenza vaccine (LAIV). Several preparations of IIVs exist on the market. The most largely used is the traditional trivalent IIV, which contains two A viruses (A H1N1 and A H3N2) and one B virus; these viruses are representative of the strains that are predicted to circulate according to the annual WHO prevision. Recently, quadrivalent preparations containing a second B virus have been licensed.^88^

Traditional trivalent IIVs can be used worldwide in all individuals starting from 6 months of age. Conversely, quadrivalent vaccines are licensed differently in various countries according to age.^89^ In the USA, a quadrivalent preparation can be given to children as young as 6 months old, whereas other quadrivalent shots are approved only for people 3 years and older.^90^ However, the policy of IIV use is strictly related to the vaccine supply. When the vaccine supply is limited in the USA, the CDC selects the population for whom the vaccines are reserved. Subjects who are considered to be at a higher risk are prioritized.^91^

Together with these basic formulations, several other IIV types are licensed. Some preparations include an increased dose of antigens, whereas in others adjuvants are added. Finally, a preparation can be administered via the intradermal route. However, these recently licensed IIVs cannot be used in children. They have been designed to overcome the main limit of the basic preparations, which is the reduced immune response in some groups, but because they are not licensed for children, they overcome that limit only for the elderly and some adults at risk. For protection in children, the LAIV has been developed in a three component formulation and, recently, in a four component formulation. Contrary to IIV, which assures protection by the induction of a vaccine strain specific antibody response and has poor efficacy against heterologous viral strains, LAIVs elicit a long-lasting, humoral and cellular response resembling natural immunity evoked after wild virus infection.^92^ Several studies conducted before licensing and in the first year after marketing have reported a greater efficacy of LAIVs compared to traditional IIVs, particularly against heterologous viral strains.^93-95^ This seems to explain why LAIV was chosen in the UK, when influenza vaccination was recommended in healthy children.^96^ In 2014, in the USA, health authorities suggested the preferential use of LAIV, when immediately available, for healthy children aged 2–8 years who did not have contraindications to the vaccine.^97^ However, national policies were reconsidered after evidence in the USA showed that LAIV efficacy in the 2013–2014 season, particularly against the A H1N1 strain, was marginal and significantly lower than that of IIV. This led the Advisory Committee on Immunization Practices (ACIP) of the USA to indicate that LAIV should not be used for the influenza season of 2016–2017.^9^ In other countries, including Canada,^98^ the UK,^99^ and Finland,^100^ LAIV was found to be less effective than expected but was more protective than in the USA. Consequently, it was not withdrawn from the list of influenza vaccines recommended for use in children by health authorities, although a continuous monitoring of LAIV efficacy was suggested.

Irrespective of its efficacy, LAIV can influence immunization policies because, contrary to traditional IIVs, it can cause clinical problems in some subjects. It is based on live attenuated viruses and cannot be administered to subjects with primary or secondary immunodeficiency. Moreover, its administration has been associated with an increased risk of hospitalization and wheezing development in children < 2 years and cannot be used in these subjects for whom only traditional IIVs are licensed.^101^

## Conclusions

National influenza immunization policies can vary significantly from country to country. These differences arise from insufficient information of the relevance of influenza infection from a clinical, social and economic point of view. Estimating the influenza disease burden has been very difficult until the beginning of this century because only recently have reliable laboratory tests capable of identifying influenza viruses among all respiratory infectious agents that can cause ILI been currently used in clinical settingsTherefore, a number of influenza cases are not detected, and the total burden of influenza is largely underestimated. The lack of precise data on the true frequency and clinical relevance of influenza infection makes it nearly impossible to establish the economic burden of influenza. Moreover, it remains very difficult to evaluate the efficacy of the different influenza vaccines and whether their use is cost-effective considering the various types of people receiving them and the indirect advantages. Fortunately, World health Organization has published a protocol to help countries estimate influenza diseases and economic burden through surveillance. Most of the disparities among countries will be overcome only when more reliable data regarding all these aspects of influenza infection, particularly those related to the true impact of the disease, are precisely defined. Only differences related to particular local situations can justify persistent different policies among countries with similar organization and quality of the health system.
